# Rapid Immunochemical Methods for Anatoxin-a Monitoring in Environmental Water Samples

**DOI:** 10.1021/acs.analchem.2c01939

**Published:** 2022-07-19

**Authors:** Ramón E. Cevallos-Cedeño, Guillermo Quiñones-Reyes, Consuelo Agulló, Antonio Abad-Somovilla, Antonio Abad-Fuentes, Josep V. Mercader

**Affiliations:** † Institute of Agricultural Chemistry and Food Technology (IATA), Spanish Scientific Research Council (CSIC), Av. Agustí Escardino 7, Paterna 46980, Valencia, Spain; ‡ Department of Organic Chemistry, University of Valencia, Doctor Moliner 50, Burjassot 46100, Valencia, Spain

## Abstract

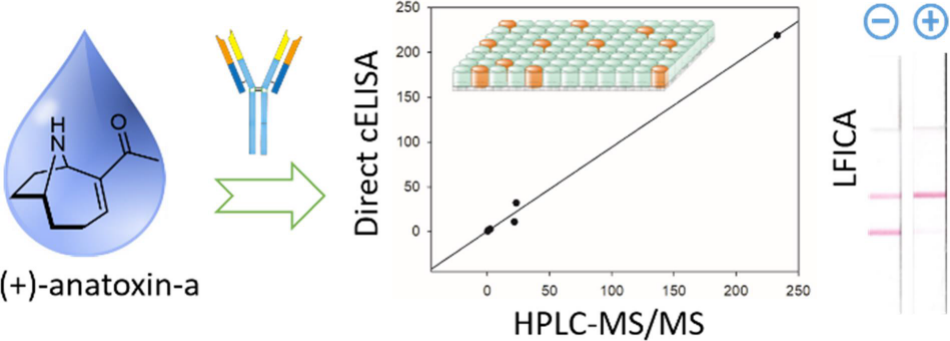

Algal blooms that contaminate freshwater resources with cyanotoxins constitute, nowadays, a global concern. To deal with this problem, a variety of analytical methods, including immunochemical assays, are available for the main algal toxins, for example, microcystins, nodularins, and saxitoxins, with the remarkable exception of anatoxin-a. Now, for the first time, highly sensitive, enantioselective immunoassays for anatoxin-a have been validated using homemade monoclonal antibodies. Two competitive enzyme-linked immunosorbent assays were developed in different formats, with detection limits for (+)-anatoxin-a of 0.1 ng/mL. Excellent recovery values between 82 and 117%, and coefficients of variation below 20%, were observed using environmental water samples fortified between 0.5 and 500 ng/mL. In addition, a lateral-flow immunochromatographic assay was optimized for visual and instrumental reading of results. This test showed a visual detection limit for (+)-anatoxin-a of 4 ng/mL. Performance with a reader was validated in accordance with the European guidelines for semiquantitative rapid methods for small chemical contaminants. Thus, at a screening target concentration of 2 ng/mL, the probability of a blank sample to be classified as “suspect” was as low as 0.2%. Finally, the optimized direct enzyme immunoassay was validated by comparison with high-performance liquid chromatography-tandem mass spectroscopy data and showed a good correlation (*r* = 0.995) with a slope of 0.94. Moreover, environmental water samples containing more than 2 ng/mL of anatoxin-a were detected by the developed dipstick assay. These results provide supplementary and complementary strategies for monitoring the presence of anatoxin-a in water.

## Introduction

Anatoxin-a is a natural, toxic alkaloid produced by several species of cyanobacteria from different genera, including *Dolichospermum* (*Anabaena*), *Aphanizomenon*, *Oscillatoria*, *Planktothrix*, and *Cylindrosperum*.^1^ These prokaryotic microorganisms can grow at vertiginous rates under specific environmental conditions, mostly in estuaries and lakes, causing sudden, massive algal proliferation events known as blooms.^2^ Nowadays, algal blooms are becoming recurrent and more intense in some regions of the world, most likely as a result of global warming and anthropogenic eutrophication,^3,4^ posing serious health risks to humans, pets, cattle, and wild animals, as well as becoming an additional economic burden on industry and public institutions.^5−8^ Anatoxin-a is one of the most frequently detected cyanotoxins in freshwater,^9^ and concentrations over 1 mg/L have been documented.^10^ Swallowing contaminated water has resulted in a large number of fatal cases in domestic animals, livestock, and wildlife.^11,12^ Humans can be exposed to anatoxin-a not only by drinking contaminated water but also by consuming algae-containing dietary supplements, or by eating fish products and bivalve mollusks—from both traditional fishing and fish farming—grown in the presence of this biotoxin.^13−17^

**Figure 1:**
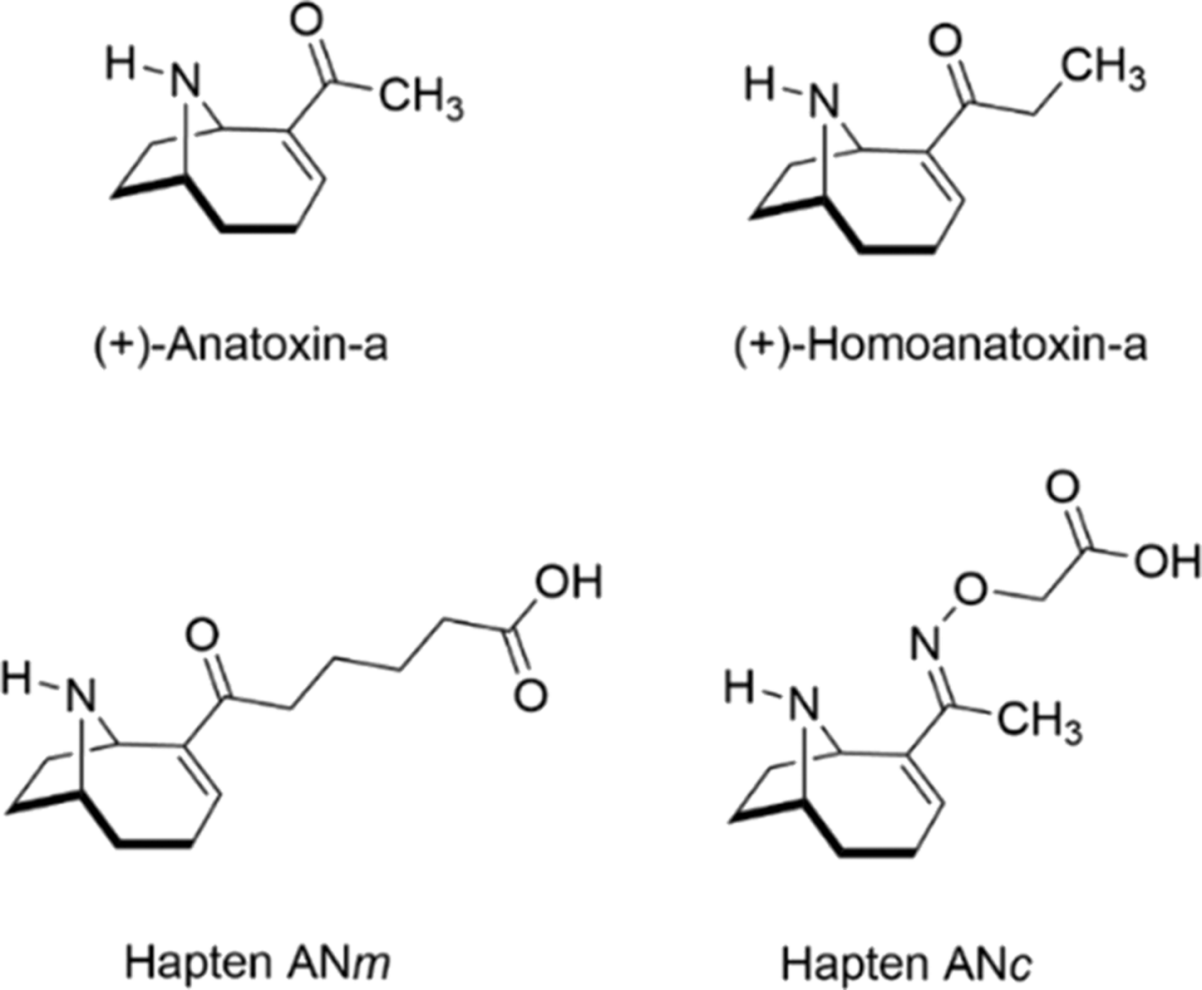
Chemical structure of (+)-anatoxin-a, (+)-homoanatoxin-a, and haptens AN*m* and AN*c*.

Because of its high toxicity in mice, the yet-unidentified anatoxin-a was initially named “Very Fast Death Factor”.^18^ The chemical structure of this very small neurotoxin was elucidated by Devlin et al. in 1977, and it was described as a bicyclic secondary amine incorporating an α,β-unsaturated ketone moiety (Figure 1).^19^ Currently, it is known that a few other algal metabolites, such as homoanatoxin-a, dihydroanatoxin-a, pinnamine, and their derivatives, possess this unusual, bicyclic homotropane chemical moiety.^20−22^ Anatoxin-a is a highly potent agonist of the nicotinic acetylcholine receptor in muscles, and because it is not degraded by acetylcholinesterase, it causes overstimulation, which may result in fatigue, convulsions, paralysis, and even death by cardiorespiratory arrest.^23^ According to toxicological and epidemiological criteria, the US EPA considered anatoxin-a a priority contaminant, thus promoting additional studies to assess the risks and establish regulations and guidelines.^24^ Currently, several US states have established maximum permitted levels (MPL) of anatoxin-a in drinking water at values that vary between 0.7 and 20 μg/L.^25^ The WHO has set provisional short-term reference values of 30 and 60 μg/L for drinking and recreational waters, respectively.^26^ Additionally, Denmark, Canada, New Zealand, and Australia have specific regulations for anatoxin-a, with provisional MPL values from 1 to 6 μg/L.^27^ Furthermore, EFSA advised that the possible presence of cyanotoxins in food should be considered as an emerging risk, and they alerted that abundant data gaps were detected, particularly on the exposure and toxicological profile of anatoxins.^28^

Several analytical methods have been developed so far for anatoxin-a detection.^29^ Formerly, gas chromatography–mass spectrometry (GC–MS) was usually employed to determine this cyanotoxin, commonly as the more volatile *N*-acetyl derivative. On the other hand, liquid chromatography methods using ultraviolet detection have low sensitivity, and derivatization is also needed. Concerning high-performance liquid chromatography-tandem mass spectroscopy (HPLC–MS/MS), interferences with phenylalanine may occur although this method does not require derivatization.^30−32^ The latter methodology is undoubtedly the most sensitive, generally accepted strategy; consequently, it was proposed by the US EPA as the official analytical method for anatoxin-a analysis in drinking water samples (EPA method 545).^33^ There is, however, a broad consensus on the need of rapid, portable, and reliable analytical methods to efficiently manage algal blooms, preventing damage to ecosystems, protecting human health, and reducing expenses.^34−36^ In this respect, biosensors using DNA aptamers specific of anatoxin-a have been published.^37,38^

Antibody-based methods to determine the main cyanotoxins, such as microcystins, saxitoxins, nodularins, and so forth, were published long ago, and an assortment of different immunoassays is commercially available, with anatoxin-a being the most relevant exception until now. Until recently, the only documented attempt to synthesize a functionalized analog of anatoxin-a with the aim of generating antibodies was published in 2009, although the authors did not demonstrate having achieved the pursued objective, nor additional results have been reported.^39^ In 2019, three functionalized derivatives of anatoxin-a were designed and prepared by our research group, and the capacity of these novel compounds to generate high-affinity antibodies when they were covalently coupled to a protein was demonstrated.^40^ Finally, five monoclonal antibodies enantiospecific of the naturally occurring (+)-anatoxin-a were raised with affinity values in the low nanomolar range. Based on these immunoreagents, the aim of the present study was to develop alternative bioanalytical methods for analyzing anatoxin-a in environmental water samples. Two enzyme-linked immunosorbent assays (ELISA) and a lateral-flow immunochromatography assay (LFICA) were optimized and characterized, and the obtained results were validated with documented concentration values from environmental samples. These tests constitute the first reported sensitive immunoassays for anatoxin-a rapid analysis.

## Experimental Section

### Reagents and Instruments

Analytical standards of both enantiomers of anatoxin-a and homoanatoxin-a (Figure 1) were synthesized in our laboratory as previously described.^41^ Concentrated stock solutions were prepared in *N*,*N*-dimethylformamide (DMF) and stored at −20 °C. Covalent conjugates of haptens AN*m* and AN*c* (Figure 1) with bovine serum albumin (BSA), ovalbumin (OVA), and horseradish peroxidase (HRP), as well as (+)-anatoxin-a enantiospecific monoclonal antibodies (mAb) were in-house-prepared in a previous study.^40^ Goat antimouse immunoglobulins polyclonal antibody (GAM) was purchased from Jackson Immunoresearch Laboratories Inc. (West Grove, PA), rabbit antimouse immunoglobulins polyclonal antibody labeled with HRP (RAM–HRP) was from Dako (Glostrup, Denmark), and GAM labeled with gold nanoparticles (GAM–GNP) was from BBI Solutions (Crumlin, UK). o-Phenylenediamine (OPD) and Biostab peroxidase conjugate stabilizer were from Merck (Darmstadt, Germany). ELISA studies were carried out in high-binding flat-bottom 96-well polystyrene Costar microplates from Corning (Corning, NY, USA). High-protein binding nitrocellulose membranes (25 mm wide and 15 μm pore size, ref. 70CNPH-N-SS40) were from MDI Advanced Microdevices PVT (Ambala Cantt, India). Cellulose sample pad (17 mm wide) and absorbent pads (43 mm wide) were from EMD Millipore Corporation (Billerica, MA) and Ahlstrom-Munksjö (Helsinki, Finland), respectively. The different parts of the strips were manually assembled using backing cards (7.8 × 30 cm) from Kenosha (Amstelveen, Netherlands). Millipore Millex-HV hydrophilic PVDF filtering devices (0.45 μm pore size) were purchased from Merck (Darmstadt, Germany).

Microplates were washed and the absorbance was read with a ELx405 washer and a PowerWave HT reader, respectively, both from BioTek Instruments (Winooski, VT, USA). A ZX1010 platform equipped with a double contact Frontline HR dispenser from BioDot (Irvine, CA) was used to functionalize the nitrocellulose membranes. The strips were cut with a CM5000 guillotine, also from BioDot. An EPSON V39 scanner was employed to scan the immunochromatography dipsticks, and RGB signals were processed using ImageJ (version 1.52a) free software.

### Competitive ELISA

For the conjugate-coated indirect cELISA format, 100 μL per well of OVA conjugate in coating buffer (50 mM carbonate–bicarbonate buffer, pH 9.6) was dispensed, and microplates were incubated overnight at room temperature. After washing the plates four times with washing solution (150 mM NaCl with 0.05% (v/v) Tween-20), the competitive reaction was carried out at room temperature during 1 h by sequentially adding 50 μL per well of standard solution or diluted sample and 50 μL per well of mAb solution. For immunoassay characterization, standards were prepared in PBS (10 mM phosphate buffer with 140 mM NaCl, pH 7.4), and the antibody was diluted in PBS-T (PBS containing 0.05% (v/v) Tween-20). For sample analysis, standards and samples were prepared in MilliQ water, whereas 2× PBS-T (20 mM phosphate buffer with 280 mM NaCl, pH 7.4, containing 0.05% (v/v) Tween-20) was employed for the mAb solution. Then, plates were washed as before, and 100 μL per well of RAM–HRP 2000-fold diluted in PBS-T was added. The amplification reaction was run during 1 h at room temperature. A final washing step was carried out, and the retained peroxidase activity was revealed with 100 μL per well of enzyme substrate solution (2 mg/mL OPD with 0.012% (v/v) H_2_O_2_ in 25 mM citrate and 62 mM phosphate buffer, pH 5.4) by incubating 10 min at room temperature. The chromogenic reaction was stopped with 100 μL per well of 1 M H_2_SO_4_, and the absorbance was immediately read at 492 nm using a reference wavelength of 650 nm.

Studies with the antibody-coated direct cELISA format were carried out using microplates precoated with 100 μL per well of a 1 μg/mL GAM solution in coating buffer by incubating overnight at 4 °C. Then, 100 μL per well of mAb solution in PBS-T was added and incubated at room temperature for 1 h. Plates were washed again, and the competitive reaction was performed by sequentially adding 50 μL per well of standard solution or diluted sample plus 50 μL per well of HRP conjugate, and incubating 1 h at room temperature. Dilution buffers were employed as indicated before for immunoassay characterization and sample analysis. After washing the plates, the color was obtained and the signal was read as described for the indirect format.

Eight-point standard curves, including a blank, were run in every microplate. A (+)-anatoxin-a concentrated solution in DMF was employed to prepare the first standard solution which was serially diluted. The measured absorbance values were fitted to a four-parameter logistic equation for standard curves using the SigmaPlot software package from SPSS (Chicago, IL). The determined half-maximum inhibitory concentration (IC_50_) and the maximum asymptote of the sigmoidal inhibition curve (*A*_max_) were considered for immunoassay characterization. Valid standard curves were those showing minimum asymptotes approaching zero, and slopes close to 1.0 were preferred. The limit of detection (LOD) was defined as the anatoxin-a concentration that reduced *A*_max_ by 10% (IC_10_). Cross-reactivity (CR) was determined as the percentage value obtained from the quotient between the IC_50_ for anatoxin-a and IC_50_ for the evaluated analyte.

### Competitive LFICA

Nitrocellulose membranes were functionalized with GAM for the control line and BSA–ANc conjugate for the test line, using 1 mg/mL solutions of these immunoreagents in 100 mM sodium phosphate buffer, pH 7.4, containing 150 mM NaCl, and by drawing the corresponding lines at 0.5 μL/cm. The control and test lines were located at 15 and 10 mm, respectively, from the base of the membrane. After dispensing, the membrane was dried with a cold air current. The sample and absorbent pads overlapped the membrane 2 and 3 mm, respectively. Finally, 4 mm strips were cut and stored at 4 °C in opaque tubes with a dry atmosphere. GAM-coated GNPs were 10-fold-diluted with 10 mM HEPES buffer, pH 7.4, and the mAb solution in Biostab (1 μg/mL) was added. The conjugation reaction was incubated for 1 h at room temperature, and then, it was supplemented with Tween-20 to a final 0.05% (v/v) concentration. The so-obtained gold-labeled antibodies (GNP–mAb) were stored at 4 °C.

The immunochromatographic assay was carried out at room temperature using 100 μL mixtures of GNP–mAb conjugate suspension and standard solution or sample dilution. This mixture was incubated 5 min at room temperature in microtiter plates, and the chromatography was run vertically by inserting the immunostrip into the microwell. Ten minutes later, the flow was stopped by removing the sample pad. The line signals were read, and the T/C value was determined as the quotient between the signal of the test (T) and control (C) lines. The inhibition ratio was calculated considering the T/C value at a particular analyte concentration and the T/C value of the blank.

Filtered anatoxin-a-free water samples were fortified with (+)-anatoxin-a. Before assaying, samples were twofold diluted with 20 mM phosphate buffer, pH 7.4, containing 100 mM NaCl and 0.05% (v/v) Tween-20. Each sample, at two spiking concentrations and a blank, was analyzed during 5 consecutive days under the same conditions in order to get 20 independent determinations for every analyte concentration. Then, the cut-off value and the false-suspect rate were determined according to the European Commission Regulation (EU) No 519/2014 for screening of mycotoxins by semiquantitative analytical methods with inversely proportional response.^42^ The cut-off value to discriminate between positive and negative samples was calculated using the following formula:
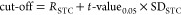
where *R*_STC_ is the average of *T*/*C* values at the screening target concentration (STC), *t*-value is the value of a one-tailed *t*-Student test for a 95% certainty—which is 1.7291 considering 19 degrees of freedom—and SD_STC_ is the standard deviation of *R*_STC_. Any sample affording a *T*/*C* value below the cut-off value will be classified as suspect to contain anatoxin-a at a concentration above the STC, assuming a 5% rate of false-negative results. To determine the probability of false-suspect results, the *t*-value was calculated as follows:
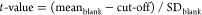
where mean_blank_ is the average of the T/C values obtained with blank samples and SD_blank_ is the corresponding standard deviation. The resulting *t*-value was used to determine the rate of false-suspect results for a one-tailed distribution using the DIST-T function of the Microsoft Excel software (Redmond, WA). The visual limit of detection (vLOD) was defined as the anatoxin-a concentration affording complete inhibition of the T signal to the naked eye.

### Analysis of Water Samples

Anatoxin-a-free water samples were collected from the nearby Túria river, Sant Vicent de Llíria lake, an irrigation channel, and a water reservoir tank from Valencia, Spain. Samples were filtered with 0.45 μm PVDF filtering devices and stored at −20 °C. Anatoxin-a-contaminated environmental water samples were kindly provided by Dr. Jutta Fastner from the German Environment Agency (UBA) in Berlin. HPLC–MS/MS analysis of samples was previously reported.^43^ Before the immunoassays were carried out, filtered samples were diluted in MilliQ water for ELISA analysis and with the above-described buffer for the LFICA determination of anatoxin-a contents.

## Results and Discussion

### Immunoreagent Selection

The linker structure and its tethering site were revealed in a previous study as key factors to generate high-affinity mAbs to anatoxin-a.^40^ No suitable antibodies could be raised from the conjugate of hapten AN*c*, whereas high-affinity binders were obtained from conjugate BSA–AN*m*. Hapten AN*m* was a perfect mimic of anatoxin-a, whereas the carbonyl group of the target compound was substituted by an oxime group in hapten AN*c*, and a shorter spacer was employed (Figure 1). Moreover, as previously described, these antibodies were enantiospecific of (+)-anatoxin-a, which is the natural isomer of this cyanotoxin.^40^

Four mAbs were evaluated using two different cELISA formats in combination with protein conjugates of haptens AN*m* and AN*c*. Concerning the direct assays, signals were only obtained with the homologous enzyme tracer (HRP–AN*m*). The shorter linker of hapten AN*c* could account for the observed absence of binding by the four assayed antibodies. In the indirect assay format, improved sensitivity was found with the heterologous conjugate OVA–AN*c*. When optimum mAb and bioconjugate concentrations were employed, all of the mAbs afforded IC_50_ values in the low nanomolar range in both assay formats (Table S1). Finally, mAbs AN*m*#38 and AN*m*#39 were selected for the development of two immunoassays with alternative formats.

### Competitive ELISA Development

Under final assay conditions, the direct and indirect cELISA showed IC_50_ values for anatoxin-a as low as 0.69 and 0.97 ng/mL, respectively (Table 1). The calculated LOD value was 0.1 ng/mL for both immunoassays. These values are comparable to those previously reported for equivalent immunoassays to other cyanotoxins typically monitored in environmental water samples, such as microcystins, nodularin, cylindrospermopsin, and saxitoxin,^44−46^ and favorably compares with aptamer-based methods for anatoxin-a.^37,38^ Moreover, the dynamic range was more than one order of magnitude wide, and the inter- and intra-day precision values were below 20 and 10%, respectively. Concerning selectivity, the optimized immunoassays showed a CR value with (+)-homoanatoxin-a (Figure 1) around 150%. Therefore, analysis of both toxins is possible with the selected immunoassays, thus broadening the applicability of the test, although this cyanotoxin is rarely found in environmental water samples from lakes with algal blooms. However, the antibodies did not significantly recognize dihydroanatoxin-a or the non-natural enantiomers (−)-anatoxin-a and (−)-homoanatoxin-a.

**Table 1:**
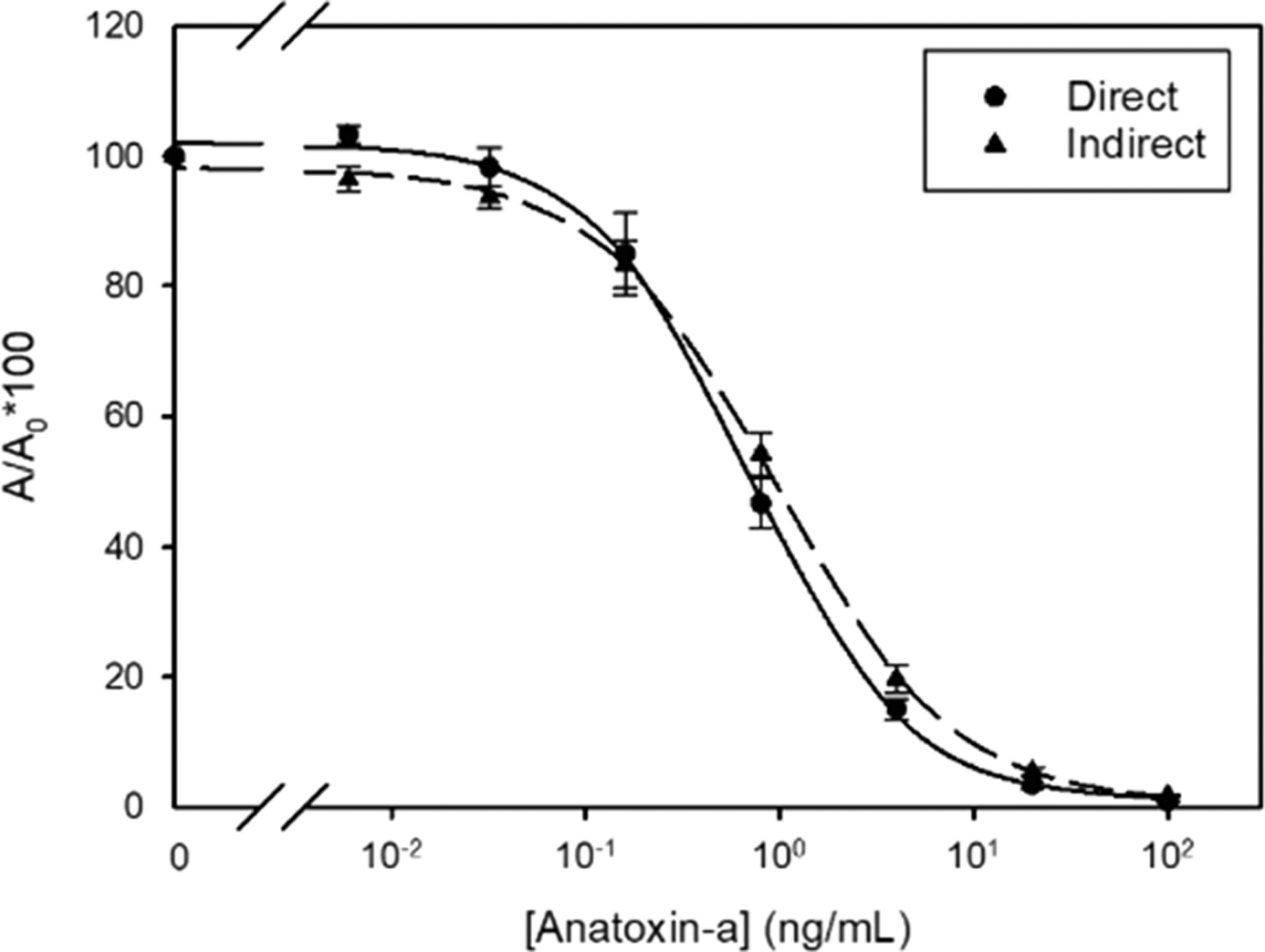
Assay Conditions and Analytical Parameters of the Optimized Immunoassays for Anatoxin-a (*n* = 4)

	direct	indirect
mAb	AN*m*#38	AN*m*#39
	500 ng/mL	25 ng/mL
conjugate	HRP–AN*m*	OVA–AN*c*
	70 ng/mL	300 ng/mL
assay buffer	10 mM phosphate, pH 7.4, 140 mM NaCl, 0.025% Tween 20	
*A*_max_	1.136 ± 0.108	1.072 ± 0.058
IC_50_ (ng/mL)	0.688 ± 0.113	0.971 ± 0.133
slope	–1.083 ± 0.127	–0.962 ± 0.083
A_min_	0.009 ± 0.013	0.003 ± 0.012
LOD (ng/mL) (IC_10_)	0.093 ± 0.033	0.099 ± 0.022
dynamic range (ng/mL)		
(IC_20_–IC_90_)	0.191–5.776	0.228–9.921
inter-day precision		
*A*_max_ (%)	9.5	5.5
IC_50_ (%)	16.5	13.7
intra-day precision		
*A*_max_ (%)	9.6	3.7
IC_50_ (%)	6.0	1.7

The influence of pH and ionic strength over the inhibition curve of both immunoassays was evaluated. Concerning the direct assay, no variation of the *A*_max_ and IC_50_ values was observed within the studied pH range—between 6.0 and 8.5 – whereas only a slight variation of these parameters was found at low NaCl concentrations (Figure S1). On the other hand, the indirect immunoassay was also robust to pH variations; however, low and high ionic strength values increased and decreased, respectively, both the *A*_max_ and IC_50_ values (Figure S2). Additionally, the influence of acetonitrile and methanol over the analytical parameters of these assays was evaluated because these are common solvents used for extraction or conditioning of environmental samples. As shown in Figures S3 and S4, increasing concentrations of acetonitrile decreased the *A*_max_ value and sharply increased the IC_50_ value of both immunoassays. On the contrary, methanol was better tolerated, particularly by the indirect assay. In summary, PBS was revealed as an optimum buffer for both immunoassays, and the concentration of organic solvents should be kept as low as possible, particularly acetonitrile.

### Competitive ELISA Performance

Water samples from different origins (river, lake, channel, and tank) were assessed for matrix effects with both of the optimized immunoassays. As depicted in Figures S5 and S6, the matrix effects were very low—no significant variation of the *A*_max_ and IC_50_ values was caused by any of the evaluated waters. In recovery studies, a wide range of anatoxin-a concentrations was studied—from 0.5 to 500 ng/mL—and accurate and precise results were observed. Recoveries from fortified samples were between 85.9% and 117.4%, and between 82.0 and 109.9%, for the direct and indirect assay, respectively (Table 2). In both cases, the CV values were below 20%. From this study, a limit of quantification (LOQ) for anatoxin-a analysis in environmental water—determined as the lowest assayed concentration affording recoveries between 80 and 120%, and CV values below 20% in spiked samples—of 0.5 ng/mL was demonstrated.

**Table 2:** Recoveries from Anatoxin-a Fortified Water Samples Analyzed by the Two Developed cELISA (*n* = 3)

		direct		indirect	
sample	[*A*]^a^	*R*^b^ (%)	CV (%)	*R*^b^ (%)	CV (%)
tank	0.5	117.4	9.9	103.8	7.8
	1.0	103.3	5.1	92.9	5.9
	2.5	98.0	9.4	95.9	8.9
	5.0	94.8	8.9	96.5	14.1
	25.0	93.1	4.6	89.6	4.8
	50.0	94.2	8.1	89.2	8.9
	100.0	89.8	5.6	90.6	12.7
	250.0	90.9	14.6	97.3	14.7
	500.0	85.9	19.7	100.2	9.9
channel	0.5	107.7	14.7	109.9	11.9
	1.0	105.3	9.8	101.1	10.5
	2.5	98.5	10.1	98.5	7.9
	5.0	95.2	9.1	99.1	8.8
	25.0	91.2	11.0	88.7	7.8
	50.0	100.9	10.1	87.3	6.0
	100.0	96.6	10.3	85.9	4.1
	250.0	95.8	11.0	91.9	2.2
	500.0	90.9	14.9	88.8	5.4
lake	0.5	106.4	15.7	101.2	9.9
	1.0	92.1	11.2	94.8	8.9
	2.5	96.3	2.9	94.4	12.3
	5.0	94.8	4.7	98.8	11.8
	25.0	96.2	8.8	82.9	8.7
	50.0	102.4	3.3	86.6	10.0
	100.0	98.8	8.6	90.2	10.9
	250.0	102.1	10.4	95.7	9.8
	500.0	100.8	13.7	98.6	3.9
river	0.5	112.3	15.4	105.9	7.4
	1.0	86.3	13.8	94.1	5.3
	2.5	99.1	11.8	92.1	7.3
	5.0	100.1	8.0	93.5	8.2
	25.0	102.2	17.2	84.1	5.4
	50.0	104.7	11.5	86.3	8.9
	100.0	97.5	6.6	82.0	11.9
	250.0	96.4	3.1	85.2	7.8
	500.0	95.3	8.7	86.9	11.4

a Analyte concentration in ng/mL. Samples spiked at 0.5–5.0 ng/mL were diluted five times while samples spiked at 25.0–500.0 ng/mL were diluted 100 times.

b Recovery values.

### Competitive LFICA Development

**Figure 2:**
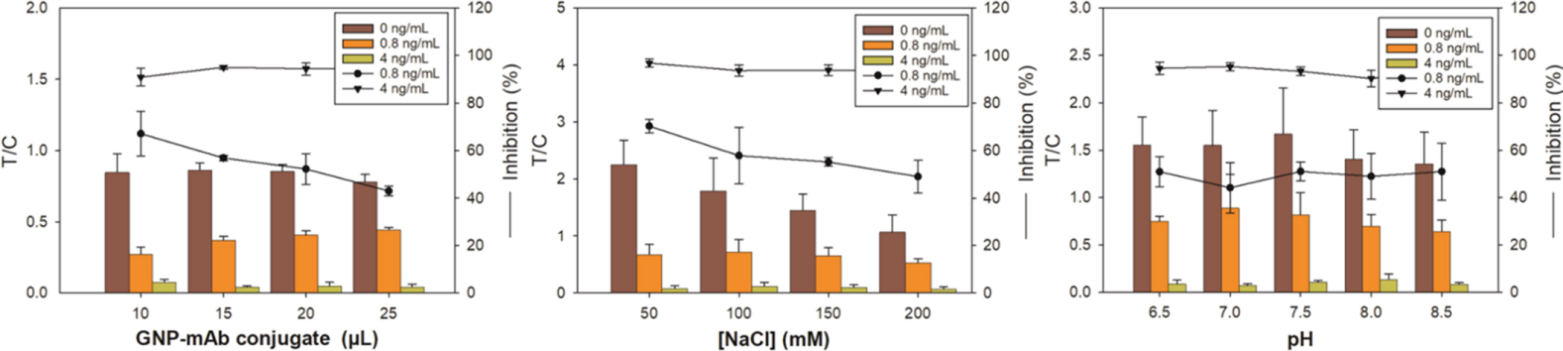
Optimization of the volume of the mAb–GNP conjugate and influence of the buffer ionic strength and pH over the studied LFICA. The final volume of the assay was always 100 μL. The T/C ratio and inhibition rate are depicted for buffer samples spiked at two anatoxin-a concentrations and a blank. On the right, an example of LFICA results is shown for buffer samples at (from left to right) 0, 0.8, and 4 ng/mL of anatoxin-a, when 20 μL of mAb–GNP and a buffer with pH 7.4 containing 50 mM of NaCl was employed.

Performance of the four available mAbs was evaluated by competitive lateral-flow assays, and antibody AN*m*#38 was selected because it provided stronger signals and superior visual sensitivity. Immunoassays were carried out using strips with conjugate BSA–AN*c* and GAM for the T and C lines, respectively, and with the mAb immobilized onto GNPs. The optimal amount of the mAb–GNP conjugate was determined. As shown in Figure 2, the T/C ratio of the blank hardly changed when different volumes of gold nanoconjugate were added; only a slight decrease was observed when 25 μL was employed. On the other hand, the inhibition ratio was higher with decreasing amounts of nanoparticles. However, the signal was too low for visual reading when 10 or 15 μL of gold bioconjugate suspension was used. For these reasons, 20 μL of the mAb–GNP conjugate was chosen as the optimum immunoreagent quantity for further assay development.

**Figure 3:**
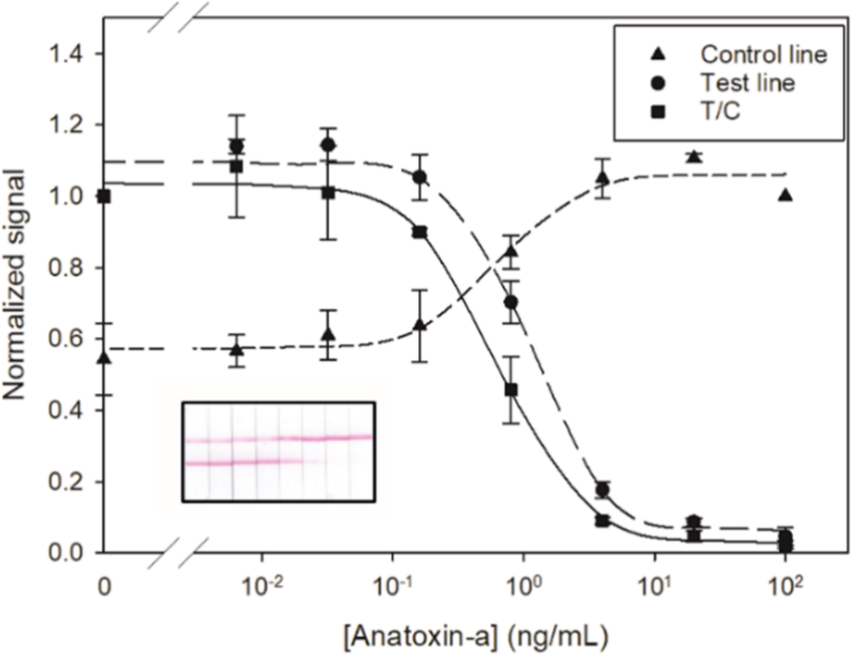
LFICA analysis of anatoxin-a standards from 6.4 × 10^–3^ to 100 ng/mL. A blank was also included (*n* = 3). The inset shows an image of the immunostrips from one replicate.

The influence of ionic strength and pH over the T/C ratio and the inhibition rate of the studied LFICA were evaluated. The highest T/C and inhibition values were observed with the lowest assayed NaCl concentration (Figure 2). Consequently, 50 mM NaCl was selected as the optimum salt concentration. Finally, no significant influence of pH was observed, so subsequent studies were carried out at pH 7.4. The signals at the test and control lines as well as the T/C ratio of the optimized assay are depicted in Figure 3 as a function of the anatoxin-a concentration. Evident signal dependency between the control and test line signals was observed. Thus, the C line was not only a control of immunostrip performance but it was also a good indicator of the antibody binding reaction with the coating conjugate and the target analyte. In fact, the sensitivity of the immunoassay was higher when the T/C value was used. Under these conditions, the calculated IC_50_ value from the T/C standard curve was 0.6 ng/mL.

### Competitive LFICA Performance

Immunoassay performance was studied according to EU guidelines for semiquantitative screening methods applied to the analysis of chemical contaminants. Four environmental water samples were spiked with anatoxin-a at 1 and 2 ng/mL, and they were analyzed during five consecutive days (Table 3).

**Table 3:**
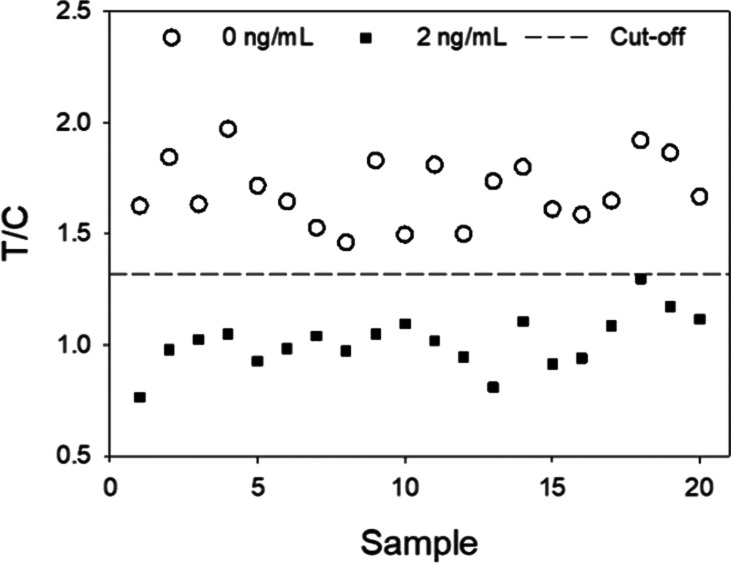
Lateral-Flow Immunochromatographic Assay Validation for Anatoxin-a Analysis in Environmental Water Samples (*n* = 20)

	STC^a^	STC
	1 ng/mL	2 ng/mL
average T/C	1.36	1.01
CV (%)	12.4	11.8
cut-off (95% certainty)	1.65	1.22
false-suspect probability (%) of blank samples	37.6	0.2
false-suspect probability (%) of samples containing 1 ng/mL		21.6

a Screening target concentration. For visual clarity, only T/C values of the blank samples and those from samples spiked at the selected STC of 2 ng/mL are depicted in the graph.

**Figure 4:**
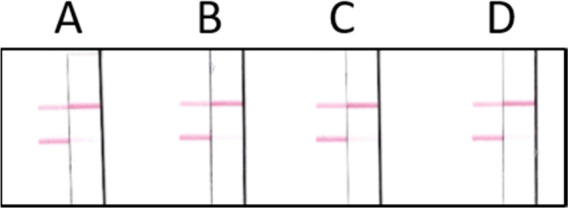
Analysis of blank (left) and anatoxin-a spiked (4 ng/mL) water (right) samples by LFICA. A: tank; B: channel; C: lake; D: river water.

Blank samples were also included in the analysis. The average T/C value and the CV for the blank samples were 1.69 and 8.7%, respectively. The average T/C values for spiked samples were 1.4 and 1.0 for the lowest and the highest anatoxin-a concentration, respectively. For these samples, the CV values were around 12%. Considering a 95% certainty level in a one-tailed t-Student distribution, the false-suspect rate for blank samples was unacceptable (38%) if an STC of 1 ng/mL was established. On the contrary, for an STC of 2 ng/mL, an excellent false-suspect rate was observed (0.2%). In the latter case, the cut-off for the T/C value was 1.22. This means that any water sample giving a T/C value of 1.22 or lower would be classified as positive with very high probability. When different fortified environmental water samples were analyzed, the developed immunoassay afforded a vLOD of 4 ng/mL for anatoxin-a (Figure 4).

### Immunochemical Analysis of Anatoxin-a in Environmental Water Samples

The direct cELISA was applied to the quantitative analysis of anatoxin-a in eight environmental water samples from lakes with blooming algae that were identified as positive by UBA. Samples were filtered and 5-fold-diluted in MilliQ water. According to the developed cELISA, the concentration of anatoxin-a in these samples was between 0.8 and 219 ng/mL (Table 4). As expected, sample 19.121 was below the LOQ when measured by the developed immunochemical method. Thus, the contents of anatoxin-a measured by direct cELISA highly correlated with the reference values. The regression analysis (*r* = 0.995) between both sets of data had an intercept value of 0.35 and a slope of 0.94 (Figure S7). The 95% confidence interval was between −5.13 and 5.81 for the intercept and between 0.87 and 1.01 for the slope, so the 0 and 1 values were included, respectively. These results show the applicability of the optimized immunochemical method for the rapid and accurate determination of anatoxin-a at very low concentration values.

**Table 4:** Anatoxin-a Concentration in Environmental Water Samples

sample	LC–MS^a^ (ng/mL)	cELISA^b^ (ng/mL)	LFICA^c^
18.027	23.2	32.0	**+**
18.044	0.5	0.8	**–**
18.045	0.5	1.0	**–**
18.056	2.3	2.7	**+**
19.109	1.4	1.9	**–**
19.121	0.3	–	**–**
19.140	233	219.1	**+**
19.141	21.7	10.8	**+**

a These values were kindly provided by UBA.

b Values are the average of three replicates.

c (+), suspect to contain more than 2 ng/mL of anatoxin-a; (−), toxin concentration below 2 ng/mL.

The same environmental water samples were analyzed by the developed immunochromatographic test for semiquantitative determination of anatoxin-a. Samples were filtered, 2-fold diluted, and incubated 5 min at room temperature with the mAb–GNP probe. Then, the immunostrip was inserted into the well, the assays were run during 10 min, and the results were read with a regular scanner. According to the developed dipstick assay, four of the samples contained anatoxin-a at concentration levels higher than 2 ng/mL, which corresponds to the expected results (Table 4), thus proving the suitability of the developed LFICA for the screening of anatoxin-a in water samples.

## Conclusions

Two complementary, highly sensitive monoclonal antibody-based analytical methods for the determination of anatoxin-a at trace levels have been developed and validated for the first time. Accurate and precise results were obtained by cELISA from recovery studies with freshwater samples spiked with this cyanotoxin. In addition, a user-friendly, point-of-need test was validated according to European guidelines for rapid, semiquantitative screening methods intended for low-molecular-weight chemical contaminants. The developed dipstick immunoassay had insignificant false-positive and false-negative rates. These results confirm that this method is suitable for monitoring programs aiming at identifying water samples contaminated with anatoxin-a at low part-per-billion levels. Furthermore, analysis of naturally contaminated environmental water samples also showed excellent correlation between levels determined by HPLC–MS/MS and the novel immunochemical assays.

## Supporting Information Available

Monoclonal antibody selection (Table S1); influence of pH, ionic strength, and solvents (Figures S1–S4); matrix effects (Figures S5–S6); and correlation between methods (Figure S7) (PDF)

## Notes

The authors declare no competing financial interest. Eurofins Abraxis, Inc. (Pennsylvania, USA; https://abraxis.eurofins-technologies.com/home) currently distributes immunostrips and ELISA kits for the detection of anatoxin-a based on the immunoreagents reported herein under a license from CSIC and UVEG.
