# Supplementary material for: Rapid Immunochemical Methods for Anatoxin-a Monitoring in Environmental Water Samples
Source: Anal Chem. 2022 Jul 19;94(30):10857–64. doi: 10.1021/acs.analchem.2c01939 (PMC9352146; doi:10.1021/acs.analchem.2c01939)
Supplement: Supplementary file 1 — ac2c01939_si_001.pdf [file ac2c01939_si_001.pdf]

SUPPORTING INFORMATION

Rapid immunochemical methods for anatoxin-a monitoring in environmental water samples

Ramón E. Cevallos-Cedeño,[1] Guillermo Quiñones-Reyes,[2] Consuelo Agulló,[2] Antonio Abad-Somovilla,[2] Antonio Abad-Fuentes,[1] Josep V. Mercader[1,*]

[1] Institute of Agricultural Chemistry and Food Technology (IATA), Spanish Scientific Research Council (CSIC), Av. Agustí Escardino 7, Paterna 46980, Valencia, Spain.

[2] Department of Organic Chemistry, University of Valencia, Doctor Moliner 50, Burjassot 46100, Valencia, Spain.

Contents	Page
Table S1	S2
Figure S1	S2
Figure S2	S2
Figure S3	S3
Figure S4	S3
Figure S5	S3
Figure S6	S4
Figure S7	S4

Table S1. Monoclonal antibody selection for direct and indirect cELISA development (n=3).[a]

	Direct			Indirect		
mAb	[mAb][b]	[HRP–AN*m*][c]	IC_{50}[d]	[mAb]	[OVA–AN*c*]	IC_{50}
AN*m*#38	500	30	3.21	100	100	3.52
AN*m*#39	300	100	4.24	25	300	3.26
AN*m*#44	300	30	3.15	50	2000	3.37
AN*m*#325	1000	30	3.50	1000	30	4.78

[a] $A_{máx}$ values were higher than 0.8. [b] Antibody concentration in ng/mL. [c] Conjugate or tracer concentrations in ng/mL. [d] Values are in nM.

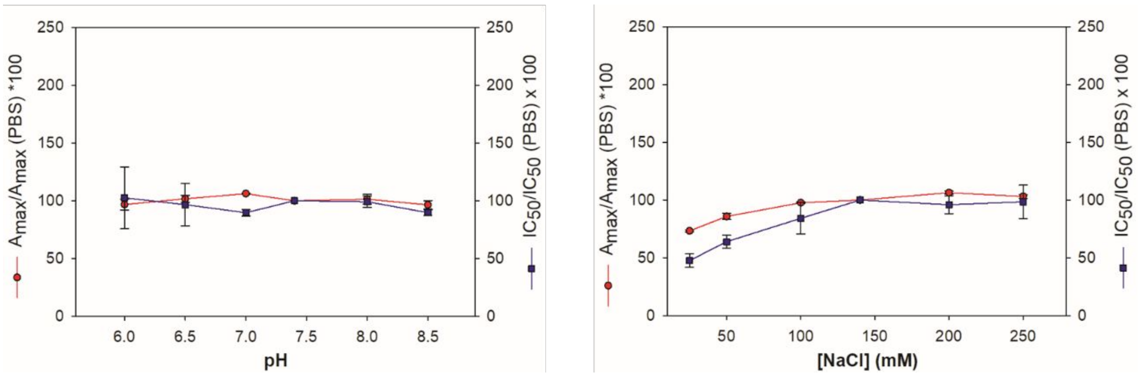

Figure S1. Influence of pH and ionic strength over the inhibition curve of the direct assay.

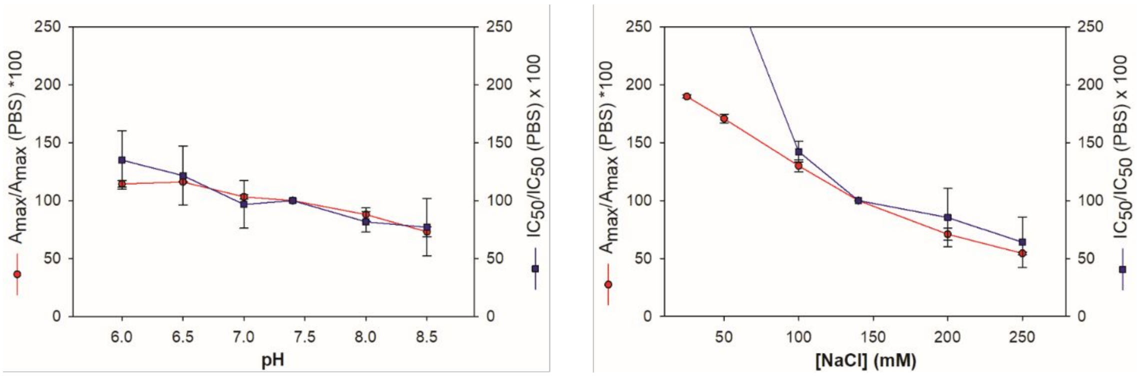

Figure S2. Influence of pH and ionic strength over the inhibition curve of the indirect assay.

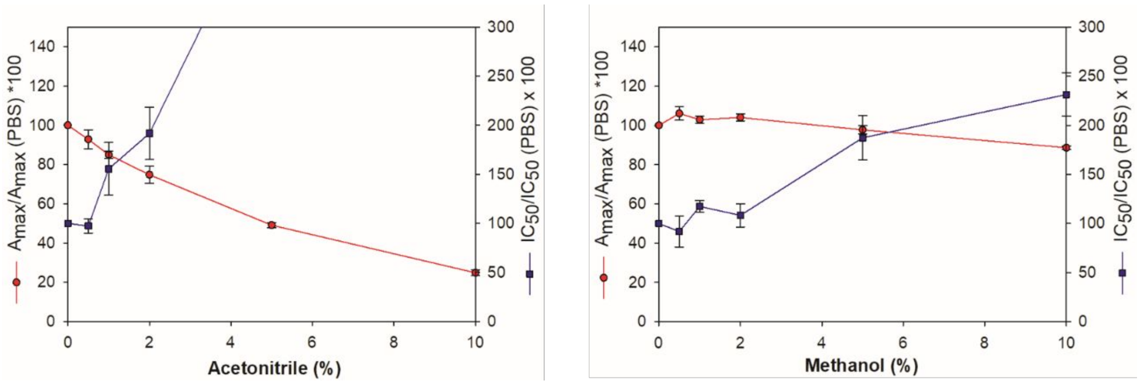

Figure S3. Influence of acetonitrile and methanol over the inhibition curve of the direct assay.

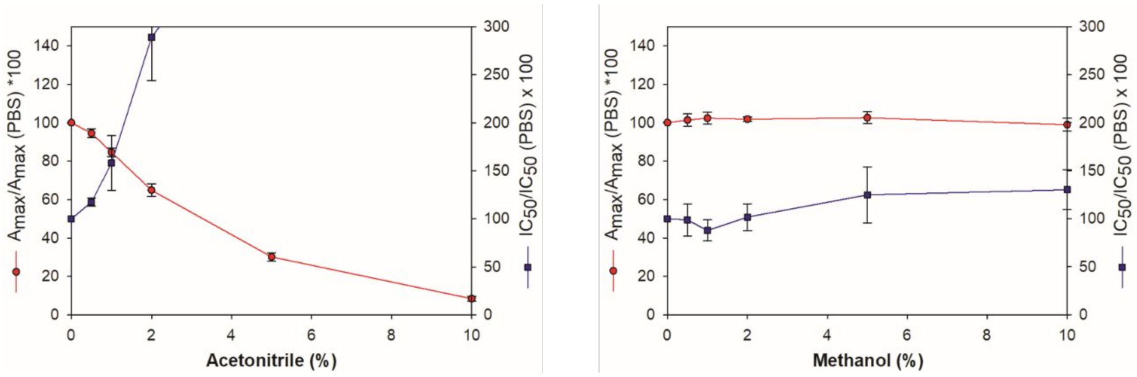

Figure S4. Influence of acetonitrile and methanol over the inhibition curve of the indirect assay.

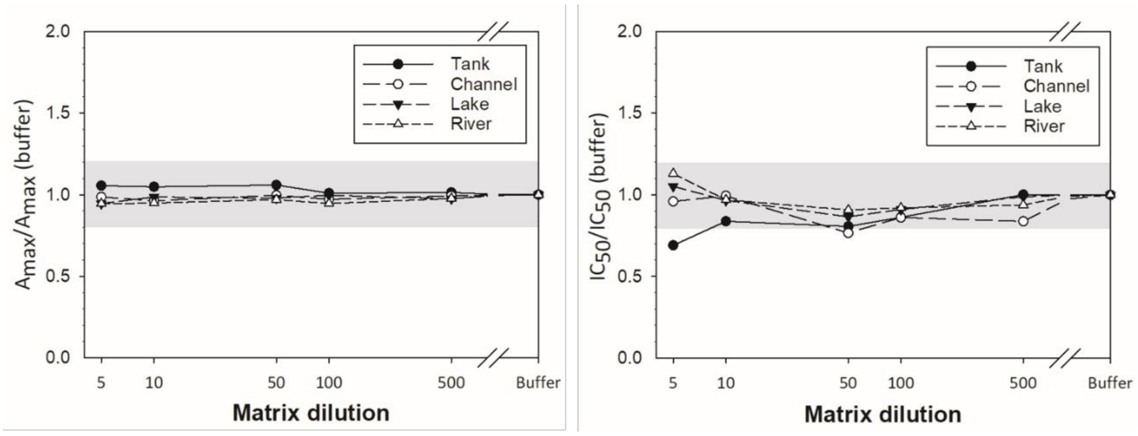

Figure S5. Matrix effect over the direct immunoassay. The gray area depicts the ±20% deviation from the inhibition curve parameters in buffer.

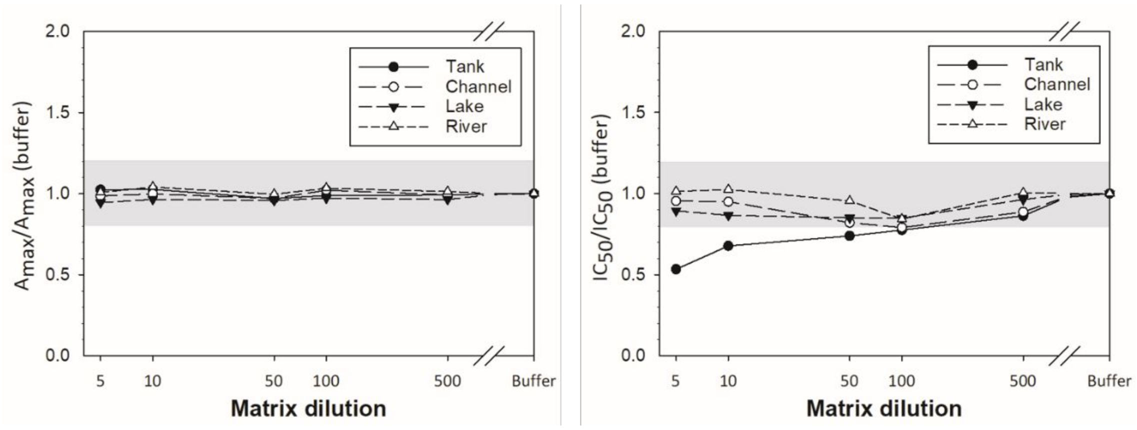

Figure S6. Matrix effect over the indirect immunoassay. The gray area depicts the ±20% deviation from the inhibition curve parameters in buffer.

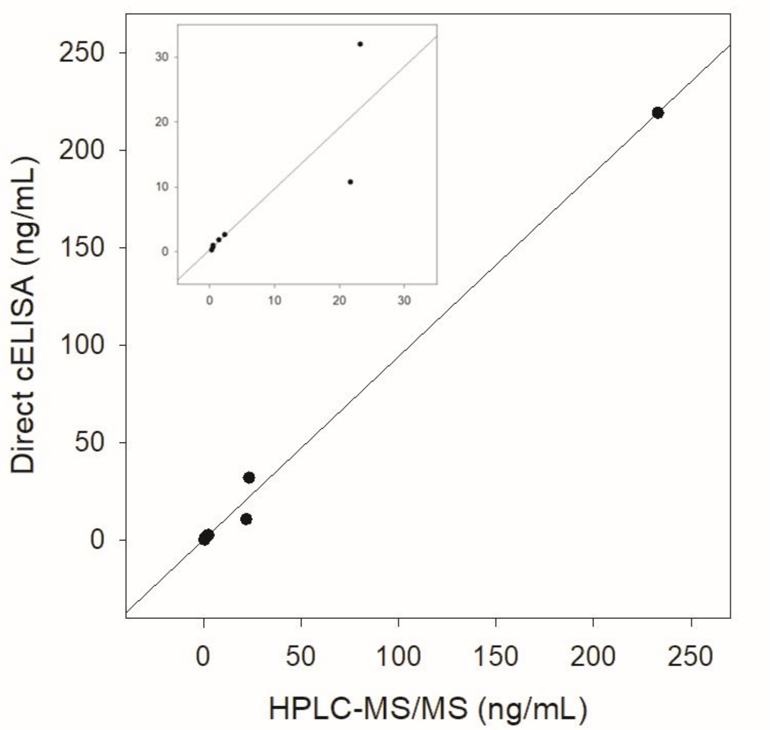

Figure S7. Validation of results from the analysis of environmental water samples by direct cELISA. Anatoxin-a reference concentration values, determined by HPLC–MS, were kindly provided by Dr. Jutta Fastner from UBA. The equation of the regression line was $y = 0.35 + 0.94x$. The insert graph shows the low concentration values.
